# Fronto-Limbic Alterations in Negatively Biased Attention in Young Adults with Subthreshold Depression

**DOI:** 10.3389/fpsyg.2017.01354

**Published:** 2017-08-07

**Authors:** Haijiang Li, Dongtao Wei, Jiangzhou Sun, Qinglin Zhang, Jiang Qiu

**Affiliations:** ^1^Department of Psychology, Shanghai Normal University Shanghai, China; ^2^Key Laboratory of Cognition and Personality (SWU), Ministry of Education Chongqing, China; ^3^Faculty of Psychology, Southwest University Chongqing, China

**Keywords:** subthreshold depression, attentional bias, attentional control, fMRI, fronto-limbic system

## Abstract

Attentional bias toward negative stimuli has been observed in major depression disorders (MDDs). Imaging studies suggest the engagement of fronto-limbic regions like amygdala, anterior cingulate cortex (ACC), and lateral prefrontal cortex, is related to negatively biased attention. However, neural correlates of attentional bias for negative stimuli in individuals with subthreshold depression (SubD), that is individuals who have clinically relevant depressive symptoms but do not fulfill the criteria for MDD, remain unclear. Here, we used functional neuroimaging and the dot-probe task to elucidate the neural substrates of negatively biased attention among individuals with SubD. Behavioral results found that individuals with SubD allocated more attention toward negative stimuli relative to neutral stimuli, which were not observed among non-depressed controls (NCs). Imaging results found greater amygdala and rostral ACC activity in attentional bias toward negative stimuli among participants with SubD compared to NCs; Additionally, participants with SubD showed reduced engagement of bilateral inferior frontal gyrus compared with NCs in the attentional processing of negative stimuli. Together, these results suggest that alteration of fronto-limbic systems relative to controls, known to be related to negative detection and attentional control, is associated with negatively biased attention in individuals with SubD.

## Introduction

Major depression disorder (MDD) is a leading cause of lifetime disability and will be the foremost leading contributor to the global burden of disease (Ferrari et al., 2013; Smith, 2014). Considering high prevalence and recurrence rate of MDD, it is critical to identify the factors that contribute to increased risk for depression (Joormann et al., 2007; Gotlib et al., 2014). Subthreshold depression (SubD), which was known as a risk factor of MDD (Cuijpers and Smit, 2004), has received considerable attention in recent years (Ma et al., 2013; Buntrock et al., 2016; Li et al., 2016). SubD is defined as individuals who have clinically relevant depressive symptoms but do not fulfill the criteria for MDD (Cuijpers and Smit, 2004; Cuijpers et al., 2007). As the prodromal phase of MDD (Eaton et al., 1995), SubD can predict the occurrence of depressive disorders within 2 years (Karsten et al., 2011). SubD, therefore, provides an ideal model for understanding the pathophysiologic mechanisms of depression and for developing preventative options for patients with MDD.

Biased attention for negative stimuli was theorized to play a vital role in the development and maintenance of depression (Beck, 2008; Disner et al., 2011). Attentional bias toward negative stimuli or away from positive stimuli were observed among both patients with MDD or participants with elevated risk for depression (Roiser et al., 2011; Gotlib et al., 2014). For example, Gotlib et al. (2004) found that depressed participants exhibited attentional bias for sad faces during the dot-probe task. Based on the mood-congruency theory, researchers found that dysphoric participants displayed more attention for negative stimuli (Koster et al., 2005). These results were also found in children of depressed mothers who were thought at elevated risk for developing a depressive disorder (Joormann et al., 2007; Gotlib et al., 2014). In addition to increased attention toward negative stimuli, depressed individuals also showed decreased attention for positive stimuli than those never depressed participants (Mogg et al., 1991; Koster et al., 2005; Kellough et al., 2008).

Neuroimaging studies revealed that biased attention toward negative stimuli mainly concerned perturbed brain activation in fronto-limbic system including the amygdala, anterior cingulate cortex (ACC) and lateral prefrontal cortex (PFC), which were regarded as playing a critical role in negative detection, attentional control and emotional regulation (Disner et al., 2011; Roiser et al., 2011; Foland-Ross and Gotlib, 2012). For instance, Chai et al. (2015) found greater amygdala activation in the processing of negative faces rather than neutral faces among high-risk individuals for depression. Attentional bias toward negative facial expressions evoked increased amygdala activity among both MDD and individuals with cognitive vulnerability (Zhong et al., 2011a). In one study which examined the neural basis of attentional bias toward sad stimuli in depressed patients using an emotional stroop task, results revealed that sad stimuli elicited elevated activity of rostral ACC among patients relative to healthy controls (Mitterschiffthaler et al., 2008). These findings were evidenced in the observation that attenuation of the amygdala and rostral ACC to negative stimuli predict later clinical response to therapy in depression (Godlewska et al., 2016).

Previous studies also found the engagement of prefrontal cortex in negatively biased attention. Participants with depression showed decreased activation of DLPFC and inferior frontal gyrus (IFG) in comparison with healthy controls during matching negative faces relative to matching stimuli shape (Zhong et al., 2011a). When exposed to negative stimuli, patients with depression expressed decreased activation of IFG and DLPFC (Fitzgerald et al., 2008). Whereas greater activation of the DLPFC were also reported in use of different emotional regulation strategy among patients with MDD (Johnstone et al., 2007; Kanske et al., 2012), suggesting its complex role in emotional processing among depressed participants. A recent study examining the stability of brain functions engaged by dot-probe task consistently observed the activation of IFG in the negative attentional bias (White et al., 2016). A intervention study explored the neural effects of cognitive training on attentional bias and found that cognitive training induced expected attentional bias and activation changes of IFG and DLPFC (Browning et al., 2010). Attentional bias modification training on participants with SubD found reduced negative attentional bias and normalized increased activation of IFG and DLPFC using resting-state functional magnetic resonance imaging (fMRI, Li et al., 2016).

The dot-probe task is regarded as a robust paradigm in detecting attentional bias of individuals with depression (Peckham et al., 2010; Dedovic et al., 2015). In this task, paired faces (either neutral-neutral, or emotional-neutral) were presented and followed by a probe replacing one of the faces in each trial. Attentional bias was quantified by subtracting the reaction time (RT) on detecting the probe replacing the emotional stimulus (congruent condition) from the RT when detecting the target replacing the neutral stimulus (incongruent condition; Bar-Haim et al., 2007). To date, imaging research on individuals with emotional disorders using the dot-probe task mainly employed two task-relevant contrasts. The first contrast compared negative incongruent trials with negative congruent trials which were thought to examine the neural activation of biased attention toward negative stimuli (Telzer et al., 2008; Dedovic et al., 2015; Fu et al., 2017). Fu et al. (2017) investigated the neural responses to the threat incongruent-versus-congruent contrast on the dot-probe task in young children with behavioral inhibition. Results found significant activation of amygdala, DLPFC, IFG, medial PFC and superior parietal lobule in response to threat incongruent relative to threat congruent trials (Fu et al., 2017). The second contrast compared all trials that include a negative face (collapsing negative incongruent trials with negative congruent trials) with neutral trials which were thought to examine the neural activation of attentional processing in the face of negative stimuli (Monk et al., 2006, 2008; Hardee et al., 2013). Monk et al. (2008) examined the neural response to the negative-versus-neutral contrast on the dot-probe task in young adults with generalized anxiety disorder (GAD) and found that young adults with GAD showed greater amygdala activation and stronger negative coupling between amygdala and IFG relative to comparison subjects when viewing negative stimuli (Monk et al., 2008).

In the current study, we aimed to explore the neural basis associated with attentional bias toward negative stimuli among participants with SubD using the dot-probe task and fMRI. Two task-relevant contrasts (negative incongruent-versus-congruent and negative-versus-neutral) were investigated for a fully understanding of neural correlates of attentional bias in relation to SubD. Based on previous studies (Monk et al., 2006, 2008; Fu et al., 2017), we hypothesized that participants with SubD will show attentional bias for negative stimuli but not for positive stimuli at the behavioral level. At the neural level, we tentatively hypothesized that participants with SubD will show greater amygdala and PFC (i.e., DLPFC and ACC) activity in response to negative incongruent-versus-congruent contrast relative to comparison participants. For the contrast of negative-versus-neutral, we expected to find decreased activation of IFG among participants with SubD relative to those healthy controls.

## Materials and Methods

### Participants

A total of 65 female undergraduate students (mean age = 20.29, *SD* = 1.24, range = 18–24) were recruited from Southwest University, Chongqing, China. Recruitment proceeded in two stages: First, a large group of participants completed the Beck Depression Inventory-II (BDI; Beck et al., 1996). From this initial group, participants who scored 14 and above or 6 and below were invited to participate in a second session within approximately 1 week. In this session, potential participants completed an in-person screening session, which included BDI and administration of the Structured Clinical Interview for DSM-IV-TR Axis I Disorders (SCID; First et al., 2001). The inclusion criteria for the study were: (1) did not fulfill the SCID diagnostic criteria for MDD; (2) had no current bipolar disorder, panic disorder or schizophrenia; (3) had no concurrent psychotherapy and psychotropic medication; and (4) not pregnant and currently not in their menstrual period.

Finally, 40 subjects with a BDI score of 14 and above at the second point in time were assigned to the SubD group, whereas 25 subjects with a BDI score of 6 and below at the second point in time were assigned to non-depressed controls (NCs). All participants were right-handed and had no life history of neurological injury or disease. This study was carried out in accordance with the approval of SWU Brain Imaging Center Institutional Ethics Review Board. In accordance with the Declaration of Helsinki (2008), written informed consent was obtained prior to engagement in the research tasks. After completing all study protocols, participants were thanked for their time and received financial compensation.

### Measures

#### Beck Depression Inventory-II

The BDI is a 21 item self-report questionnaire measuring severity of depressive symptoms during the past week on a four-point scale (0–3), with higher scores indicating more severe symptomatology (Beck et al., 1996). The BDI is considered to be a valid and reliable measure assessing the severity of depressive symptoms in clinical and non-clinical samples.

#### Dot-Probe Task

The dot-probe task was used to measure attentional bias toward negative stimuli (MacLeod et al., 2002) of individuals with SubD or NC. In this task, positive, neutral and negative faces (positive: happy faces; neutral: neutral faces; negative: sad and angry faces) were selected from the NimStim Face Stimulus Set (Tottenham et al., 2009) to create positive-neutral, negative-neutral, and neutral-neutral pairs. Independent ratings of the facial expressions (*n* = 23 participants) on a seven point scale ranging from 1 (negative) to 7 (positive) confirmed a significant difference in valance *F*(2,44) = 576.12, *p* < 0.001, (M ± SD, negative: 1.69 ± 0.15; happy: 5.95 ± 0.66; neutral: 4.03 ± 0.30). There was no significant difference in valance between angry and sad facial expressions for negative stimuli *t*(22) = 0.72, *p* = 0.48, (M ± SD, angry: 1.65 ± 0.31; sad: 1.73 ± 0.24).

Each trial started with a 500 ms fixation cross (gray background), followed by the face pair stimuli (visual angle of each stimuli ≈5.8°× 6.5°, vertical separation between the center of each stimuli ≈11°) for 500 ms which was replaced with a pair of dots (i.e., **:** or..) in the spatial location previously occupied by one of the stimuli. Participants were instructed to press one of two buttons to indicate the type of dot-probe (i.e., horizontal or vertical) as quickly and as accurately as possible. The probes were presented until 1100 ms had elapsed. The inter-trial interval was 1900 ms. Before scanning, participants were trained on the task.

The task involved a total of 200 trials divided across two blocks. There were 32 trials for each of the five conditions (negative congruent, NeC; negative incongruent, NeIC; positive congruent, PoC; positive incongruent, PoIC; and neutral-neutral, Neutral trials). In order to keep the same number of trials for each condition, sad and angry trial types were combined together to get 32 trials in negative congruent or incongruent condition. An additional 40 blank trials (no faces and no dots) were presented to serve as the implicit baseline. In both groups, the positive and negative faces appeared randomly and equally on either the upper or lower location of the screen.

A measure of attentional bias toward emotional stimuli was calculated separately for positive and negative stimuli by subtracting the mean RT on emotional congruent (e.g., NeC) trials from emotional incongruent (e.g., NeIC) trials. Positive scores reflect a biased attention toward emotional stimuli and negative scores reflect a biased attention away from emotional stimuli. In the calculation of attention bias scores, inaccurate trials, trials with RT less than 150 ms or more than 1100 ms were excluded from analyses. Additionally, for each participant, trials with more than 2.5 standard deviations of the mean RT for each condition were also excluded (e.g., Britton et al., 2015; Naim et al., 2015).

### Image Acquisition

MR images were acquired with a 3T Siemens Trio MRI scanner with an 8-channel head coil (Siemens Medical, Erlangen, Germany). T2-weighted gradient echo planar imaging (EPI) were used to obtain the functional images (TR/TE = 2000 ms/30 ms; Flip angle = 90°; Slices = 32; Field of view = 220 mm × 220 mm; matrix size = 64 × 64; Voxel size = 3.4 mm × 3.4 mm × 4 mm). High resolution T1-weighted anatomical images were also recorded for anatomical reference (TR/TE = 1900 ms/2.52 ms; Flip angle = 9°; Slices Thickness = 1 mm; Slices = 176; Resolution matrix = 256 mm × 256 mm; Voxel size = 1 mm × 1 mm × 1 mm).

### Image Processing and Analyses

SPM8 were used to preprocess the functional images (Wellcome Department of Cognitive Neurology, London, United Kingdom^1^), including slice timing correction, motion correction, and normalization into EPI templates based on the MNI space (voxel size was resampled into a 3 mm × 3 mm × 3 mm). Then, images were smoothed with a 6-mm full width half-maximum Gaussian kernel to increase signal-to-noise ratio.

In the first level analysis, single subject statistical parameter maps were created for each of the five conditions (NeC, NeIC, PoC, PoIC, Neutral) using the general linear model. Blank trials were modeled as an implicit baseline. An additional non-interest regressor modeled excluded nuisance (incorrect, out-of-range, and null response) trials. In addition, the movement correction parameters were added as covariates of no interest to control for possible contribution of movement to the changes in brain activity. Contrast coefficients were calculated at the first level based on comparisons of coefficients for specific conditions, and were submitted to group-level random-effect analysis to estimate error variance across individuals.

The main contrasts of interest were NeIC > NeC trials and Negative (collapsed NeIC trials with NeC trials) > Neutral (neutral-neutral) trials, which reflected the brain activation when participants showed attentional bias for negative and brain responses following negative stimuli distraction respectively (White et al., 2016). For comparison, PoIC-versus-PoC and Positive (collapsed PoIC trials with PoC trials) versus Neutral were examined as well.

Given theoretical precedent, *a priori* structural region of interest (ROI) analyses were conducted to look for significant interactions in amygdala, ACC, IFG and DLPFC during negative attentional bias. The Wake Forest University (WFU) Pick Atlas (Maldjian et al., 2003) was used to define these structural ROIs: Amygdala, ACC, IFG (inferior frontal opercularis, inferior frontral triangularis, and inferior frontal orbitalis), and DLPFC (BA9, BA46). Two-sample *t*-tests were used to determine regional group differences at the second level analyses. Overall significance was achieved with a threshold of *p* < 0.05 with family-wise error (FWE) small volume correction (SVC) at the cluster level within each ROI in combination with an underlying voxel level threshold of *p* < 0.001, uncorrected.

## Results

### Behavioral Results

Participants in the SubD and NCs did not differ in age, *t*(63) = 0.16, *p* = 0.88; however, they differed in BDI scores, *t*(63) = 12.63, *p* < 0.01, **Table 1**. A repeated measures ANOVA on Group (SubD, NCs) × Attentional bias score (Negative, Positive) revealed that the two way interaction of group × Attentional bias score was not significant, *F*(1,63) = 2.54; *p* = 0.12. Main effects of group and attentional bias scores were not significant as well, *ps* > 0.1. Considering the attentional bias score reflects attention bias for positive or negative stimuli relative to neutral stimuli, follow-up one sample *t*-tests were conducted separately for those with SubD and NCs to determine whether the bias scores of each group showed a significant degree of laterality. The results showed that the attentional bias score for negative stimuli was significantly higher than zero for those with SubD, *t*(39) = 3.28, *p* < 0.01, whereas the attentional bias score for negative stimuli did not differ from zero for those with NCs, *t*(39) = 0.67, *p* > 0.1. No group difference was found for attentional bias to negative or positive stimuli (*p*s > 0.1). No significant effects were found on the bias score for positive stimuli.

**Table 1:** Descriptive statistics for participants with subthreshold depression and non-depressed control.

	Subthreshold depression		Non-depressed			
	*Mean*	*SD*	*Mean*	*SD*	*t*	*p*
Age	20.28	0.85	20.32	1.46	0.14	0.89
BDI-II	24.23	8.06	3.40	2.06	15.55	0.01
Negative bias score (ms)	9.86	19.02	2.25	30.93	1.23	0.22
Positive bias score (ms)	2.60	24.41	10.10	29.58	1.11	0.27

### Imaging Results

**FIGURE 1:**
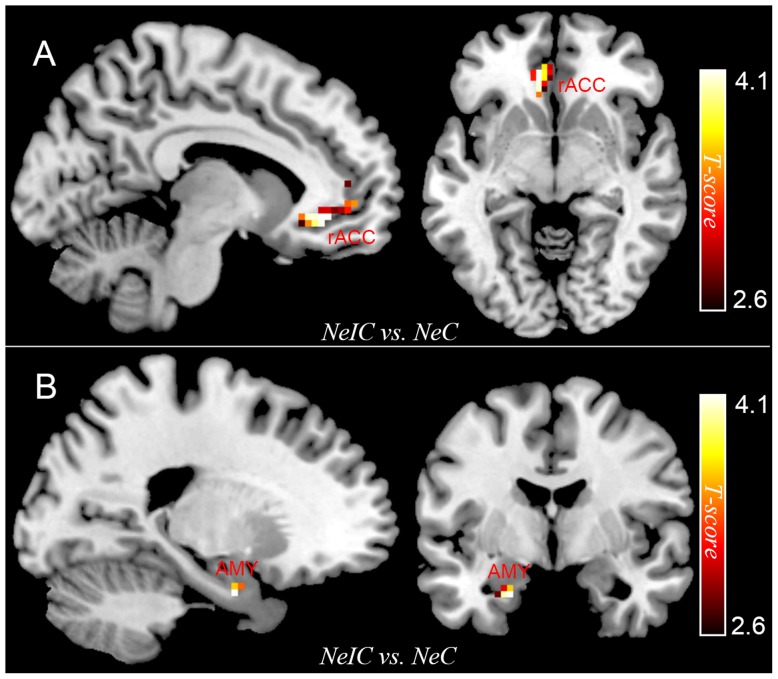
The differential activation between participants with subthreshold depression (SubD) and those with non-depressed control (NC) in the contrast of negative incongruent trials versus negative congruent trials. Participants with SubD exhibited greater activation in the left amygdala **(A)** and left rostral ACC **(B)** than the NCs. The results are shown with *p* < 0.005 uncorrected for visualization purposes.

Differential activation between participants with SubD and NCs was examined for the contrast of NeIC-versus-NeC. The results showed that participants with SubD exhibited greater activation in the left amygdala (peak MNI coordinate: -21, -3, -27; *t* = 4.12, *p*(corrected) < 0.05; *Cluster size* = 135 mm^3^; **Figure 1B** and **Table 2**) and left rostral ACC (peak MNI coordinate: -9, 36, -6; *t* = 4.03, *p*(corrected) < 0.05; *Cluster size* = 378 mm ^3^; **Figure 1A** and **Table 2**) than the NCs. No other significant effects were found in this contrast. No significant effects were found in these regions in the contrasts of PoIC versus PoC trials.

**Table 2:** Brain regions exhibiting significant activity among participants with subthreshold depression.

Brain regions	Side	MNI coordination			Cluster size (mm^3^)	Peak *T*-Value^a^	*p^b^*
		*x*	*y*	*z*			
**NeIC versus NeC**							
rACC	L	-9	36	-6	378	4.03	0.015
Amygdala	L	-21	-3	-27	135	4.12	0.002
**Negative versus Neutral**							
IFG	L	-39	18	12	2079	-5.38	0.001
IFG	R	42	27	0	1728	-4.73	0.006

*IFG, inferior frontal gyrus; rACC, rostral anterior cingulate cortex; NeIC, negative incongruent; NeC, negative congruent. ^a^A positive *t*-value indicates increased activity, a negative *t*-value indicates decreased activity. ^b^corrected *p*-value*.

**FIGURE 2:**
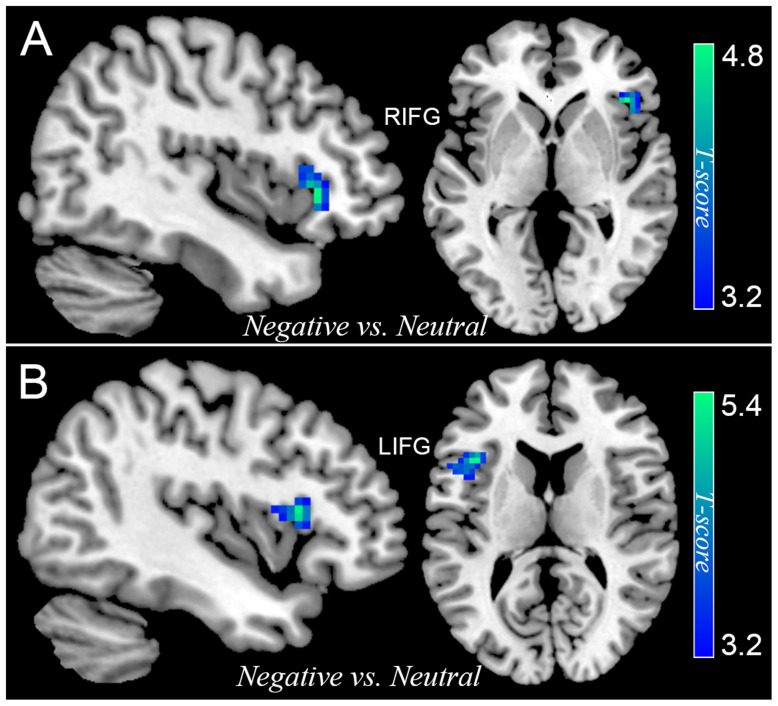
The differential activation between participants with SubD and those with NC in the contrast of negative trials (collapse negative incongruent trials with negative congruent trials) versus neutral trials. Participants with SubD exhibited lower activation in the right IFG **(A)** and left IFG **(B)** than the NCs. The results are shown with *p* < 0.05 after correction for multiple comparisons.

Differential activation between participants with SubD and NCs was also examined for the contrast of all negative trials (collapsed NeIC trials with NeC trials) versus neutral trials. Participants with SubD showed reduced activation of right IFG (peak MNI coordinate: 42, 27, 0; *t* = -4.73, *p*(corrected) < 0.05; *Cluster* *size* = 2376 mm^3^; **Figure 2A** and **Table 2**) and left IFG (peak MNI coordinate: -39, 18, 12; *t* = -5.38, *p*(corrected) < 0.05; *Cluster size* = 2187 mm^3^; **Figure 2B** and **Table 2**) than the NCs. No other significant effects were found in this contrast. No significant effects were found in these regions in the contrasts of PoIC versus PoC trials.

### Relationship between Brain Activity and Depression Severity

Correlations between brain activation from NeIC-versus-NeC and negative-versus-neutral contrasts and depression severity of participants with SubD were also explored. This revealed a negative and significant relationship between BDI scores and left IFG activity (*r* = -0.37, *p* = 0.02) and a marginal negative significant correlation between BDI scores and right IFG activity (*r* = -0.3, *p* = 0.06). However, there was no significant relationship between depression severity and brain activity of amygdala and ACC among participants with SubD.

## Discussion

The current study aimed to investigate the neural responses associated with attentional bias for negative stimuli among young adults with SubD using the dot-probe task and functional MRI. Two task-relevant contrasts of negative incongruent-versus-congruent trials or negative-versus-neutral trials were both assessed. Consistent with our hypothesis, behavioral results revealed that individuals with SubD exhibited an attentional bias toward negative stimuli in comparison with NCs. Imaging results found greater activation of amygdala and rostral ACC in the contrast of negative incongruent-versus-congruent trials among participants with SubD rather than NCs. In the contrast of negative-versus-neutral contrast, participants with SubD showed less recruitment of bilateral IFG compared with NCs. Together, these findings suggest that participants with SubD exhibited negative biased attention and fronto-limbic alterations associated with attentional bias for negative stimuli may contribute to increased risk for depression.

Participants with SubD allocated more attention toward negative stimuli than neutral stimuli, while NCs did not show significant attentional bias for negative or positive stimuli. The interaction between negative emotions and attention has been examined by a lot of studies in healthy participants (Vuilleumier et al., 2001; Pessoa et al., 2002), patients with attentional deficits (Tamietto et al., 2015), or other psychiatric disorders (Roiser et al., 2011; Gotlib et al., 2014). Previous studies on both depressed patients or individuals with high risk for depression consistently observed biased attention for negative stimuli (Roiser et al., 2011; Gotlib et al., 2014). This is grounded by a recent meta-analysis on attentional bias in depressed individuals which revealed the existence of negatively biased attention in depression (Peckham et al., 2010). These results support the cognitive conceptualizations of depression proposing that negative bias is a trait characteristic of depression vulnerability (Beck, 2008; Disner et al., 2011), however, the fact that negative mood may also contribute to negative bias cannot be excluded.

Participants with SubD showed greater amygdala and rostral ACC activity for negative stimuli than NCs in the contrast of negative incongruent-versus-congruent. These findings are consistent with previous studies which observed increased amygdala activity in response to negative information in depressed patients (Lévesque et al., 2003; Siegle et al., 2007; Greening et al., 2013) or participants with elevated risk for depression (Zhong et al., 2011b; Albert et al., 2016). The amygdala has previously been implicated in enhanced perception of emotionally salient events (i.e., aversive stimuli, Anderson and Phelps, 2001). Participants with remitted depression showed greater attentional bias for negative stimuli and increased amygdala activity for negative images than healthy subjects (Albert et al., 2016). In comparison with neutral faces, presentation of sad faces were associated with significant activation of bilateral amygdala among depressed patients during an affective detection task (Dannlowski et al., 2007). Patients with depression expressed enhanced amygdala activation for negative faces that were the target of attention, but not when negative faces were unattended distracters (Greening et al., 2013). Consistent with a recent meta-analysis of negatively attentional bias of MDD (Hamilton et al., 2012), increased amygdala activation in attentional bias for negative stimuli may suggest a maladaptive detection of negative information in vulnerable individuals of depression.

The region of the rostral ACC is regarded as an affective division of cingulate cortex and plays a vital role in affective processing and cognitive control (Bush et al., 2000; Shackman et al., 2011). A recent review indicated that altered rostral ACC activity contributes to biased attention for negative information in depression by disrupting efficient inhibition of negative stimuli (Disner et al., 2011). Such results are consistent with previous findings that depressed patients recruit more rostral ACC during sad stimuli processing compared with healthy controls, and this enhanced activation of rostral ACC positively correlates with response time to negative stimuli in depressed patients (Mitterschiffthaler et al., 2008). Researchers also found increased functional connectivity between rostral ACC and amygdala in the processing of negative self-related information in depressed individuals in comparison with control participants (Yoshimura et al., 2010). In addition, an imaging study on attentional training in healthy participants found significant training effects in rostral ACC when conflicting demands were made on attention (Browning et al., 2010). Thus, the results suggest that participants with SubD may need more cognitive effort to disengage attention from negative stimuli.

In the present study, participants with SubD exhibited hypoactivation in bilateral IFG when distributing more attention toward negative stimuli relative to neutral compared with NCs. The IFG is considered to be involved in inhibitory control (Aron et al., 2014), which stems from the active maintenance of dynamic patterns in this region to achieve them (Miller and Cohen, 2001). A weakness of the IFG will affect many types of inhibitory control including attentional control in neuropsychiatric disorders (Aron et al., 2014). Indeed, participants with depression exhibited decreased activation in the IFG in a response inhibition task (Townsend et al., 2012), emotional regulation task (Greening et al., 2014), and a negative facial expression matching task compared to healthy subjects (Zhong et al., 2011a). Activation of the IFG were associated with reduced emotional responses through an attentional bias mechanism that acts on the subcortical region, such as amygdala (Wager et al., 2008; Beevers et al., 2010). This is consistent with prior findings that interaction between amygdala and prefrontal regions like ACC, IFG, and DLPFC played an important role in regulating emotional faces processing (Lapate et al., 2016; Diano et al., 2017). Decreased activity in the IFG combined with altered patterns of rostral ACC activity is thought to conduce to rumination by facilitation the inhibition of positive information and the impedance of the inhibition of negative information (Elliott et al., 2002). Our finding of decreased recruitment of the IFG for individuals with SubD in the presence of negative relative to neutral stimuli suggests that, compared with healthy controls, individuals with SubD may exhibit an inefficient attentional disengagement from negative stimuli which is related to decreased activity in the IFG.

Notwithstanding its potential implications, some limitations of this study should be acknowledged. First, there are discrepancies between increased and decreased activation of the IFG in studies on emotional processing in depressed individuals (Diener et al., 2012; Miller et al., 2015). In the present study, we argued that the decreased activation of IFG in response to negative stimuli is due to response inhibition deficits among participants with SubD. Whereas ignoring negative faces compared to neutral faces, depressed patients displayed increased IFG responses compared to healthy controls (Colich et al., 2017). Researchers suggest that this discrepancy of the functional differences among PFC may be sensitive to task demands, with increased IFG activity reflecting the need for more cognitive resources in depressed individuals, whereas decreased IFG activation reflecting the dysfunction in response inhibition (Aron et al., 2014; Colich et al., 2017). Second, only young women with SubD were recruited in the current study, which may limit the generalizability of the findings to men. However, previous studies have reported that both depression and anxiety disorders are more prevalent among women than among men (Kessler et al., 1994). Thus, we decided to recruit only female participants to reduce the heterogeneity of our sample. Future research should examine sex differences in neural associations of negatively attentional bias. Finally, the timing issue is an important influence factor in the research of interaction between emotion and attention (Costa et al., 2014). However, the lower temporal resolution of fMRI made it non-optimal to explore the directionality of the interaction of amygdala and prefrontal regions in the processing of emotion and attention (Eger et al., 2003; Esslen et al., 2004). Future studies should examine this issue using higher temporal resolution equipment like EEG with source analysis (Costa et al., 2014).

## Conclusion

The current study found that individuals with SubD allocated more attention toward negative stimuli, and that aberrant fronto-limbic regions were engaged in negatively biased attention. These findings suggested that participants with SubD may exhibit impaired ability in the negative detection and inhibitory control in response to negative information. Therefore, negative biased attention could be considered as an early intervention target for those individuals at a risk of developing major depressive disorder.

## Author Contributions

Conceived and designed the experiments: HL, DW, JS, QZ, and JQ. Collected and Analyzed the data: HL, DW, and JS. Contributed to the writing of the manuscript: HL, DW, QZ, and JQ.

## Conflict of Interest Statement

The authors declare that the research was conducted in the absence of any commercial or financial relationships that could be construed as a potential conflict of interest.

## Footnotes

1 http://www.fil.ion.ucl.ac.uk/spm/software/spm8/

## References

[B1] Albert K. Gau V. Taylor W. D. Newhouse P. A. (2016). Attention bias in older women with remitted depression is associated with enhanced amygdala activity and functional connectivity. *J. Affect. Disord.* 210 49–56. 10.1016/j.jad.2016.12.010 28012352 PMC5292067

[B2] Anderson A. K. Phelps E. A. (2001). Lesions of the human amygdala impair enhanced perception of emotionally salient events. *Nature* 411 305–309. 10.1038/35077083 11357132

[B3] Aron A. R. Robbins T. W. Poldrack R. A. (2014). Inhibition and the right inferior frontal cortex: one decade on. *Trends Cogn. Sci.* 18 177–185. 10.1016/j.tics.2013.12.003 24440116

[B4] Bar-Haim Y. Lamy D. Pergamin L. Bakermans-Kranenburg M. J. van Ijzendoorn M. H. (2007). Threat-related attentional bias in anxious and nonanxious individuals: a meta-analytic study. *Psychol. Bull.* 133 1–24. 10.1037/0033-2909.133.1.1 17201568

[B5] Beck A. T. (2008). The evolution of the cognitive model of depression and its neurobiological correlates. *Am. J. Psychiatry* 165 969–977. 10.1176/appi.ajp.2008.08050721 18628348

[B6] Beck A. T. Steer R. A. Brown G. (1996). *Manual for the Beck Depression Inventory-II.* San Antonio, TX: Psychological Corporation.

[B7] Beevers C. G. Pacheco J. Clasen P. McGeary J. E. Schnyer D. (2010). Prefrontal morphology, 5-HTTLPR polymorphism and biased attention for emotional stimuli. *Genes Brain Behav.* 9 224–233. 10.1111/j.1601-183X.2009.00550.x 20039945 PMC2962851

[B8] Britton J. C. Suway J. G. Clementi M. A. Fox N. A. Pine D. S. Bar-Haim Y. (2015). Neural changes with attention bias modification (ABM) for anxiety: a randomized trial. *Soc. Cogn. Affect. Neurosci.* 10 913–920. 10.1093/scan/nsu141 25344944 PMC4483563

[B9] Browning M. Holmes E. A. Murphy S. E. Goodwin G. M. Harmer C. J. (2010). Lateral prefrontal cortex mediates the cognitive modification of attentional bias. *Biol. Psychiatry* 67 919–925. 10.1016/j.biopsych.2009.10.031 20034617 PMC2866253

[B10] Buntrock C. Ebert D. D. Lehr D. Smit F. Riper H. Berking M. (2016). Effect of a web-based guided self-help intervention for prevention of major depression in adults with subthreshold depression: a randomized clinical trial. *JAMA* 315 1854–1863. 10.1001/jama.2016.4326 27139058

[B11] Bush G. Luu P. Posner M. I. (2000). Cognitive and emotional influences in anterior cingulate cortex. *Trends Cogn. Sci.* 4 215–222. 10.1016/S1364-6613(00)01483-2 10827444

[B12] Chai X. J. Hirshfeld-Becker D. Biederman J. Uchida M. Doehrmann O. Leonard J. A. (2015). Functional and structural brain correlates of risk for major depression in children with familial depression. *Neuroimage* 8 398–407. 10.1016/j.nicl.2015.05.004 26106565 PMC4474282

[B13] Colich N. L. Ho T. C. Foland-Ross L. C. Eggleston C. Ordaz S. J. Singh M. K. (2017). Hyperactivation in cognitive control and visual attention brain regions during emotional interference in adolescent depression. *Biol. Psychiatry* 2 388–395. 10.1016/j.bpsc.2016.09.001 PMC5586219 28890942

[B14] Costa T. Cauda F. Crini M. Tatu M. Celeghin A. de Gelder B. (2014). Temporal and spatial neural dynamics in the perception of basic emotions from complex scenes. *Soc. Cogn. Affect. Neurosci.* 9 1690–1703. 10.1093/scan/nst164 24214921 PMC4221209

[B15] Cuijpers P. Brannmark J. van Straten A. (2007). Psychological treatment of postpartum depression: a meta-analysis. *J. Clin. Psychol.* 64 103–118. 10.1002/jclp.20432 18161036

[B16] Cuijpers P. Smit F. (2004). Subthreshold depression as a risk indicator for major depressive disorder: a systematic review of prospective studies. *Acta Psychiatr. Scand.* 109 325–331. 10.1111/j.1600-0447.2004.00301.x 15049768

[B17] Dannlowski U. Ohrmann P. Arolt V. Heindel W. Kersting A. Baune B. T. (2007). Amygdala reactivity to masked negative faces is associated with automatic judgmental bias in major depression: a 3 T fMRI study. *J. Psychiatry Neurosci.* 32 423–429. 18043766 PMC2077348

[B18] Dedovic K. Giebl S. Duchesne A. Lue S. D. Andrews J. Efanov S. (2015). Psychological, endocrine, and neural correlates of attentional bias in subclinical depression. *Anxiety Stress Coping* 29 479-96. 10.1080/10615806.2015.1101457 26419271

[B19] Diano M. Tamietto M. Celeghin A. Weiskrantz L. Tatu M.-K. Bagnis A. (2017). Dynamic changes in amygdala psychophysiological connectivity reveal distinct neural networks for facial expressions of basic emotions. *Sci. Rep.* 7:45260 10.1038/srep45260 PMC5366904 28345642

[B20] Diener C. Kuehner C. Brusniak W. Ubl B. Wessa M. Flor H. (2012). A meta-analysis of neurofunctional imaging studies of emotion and cognition in major depression. *Neuroimage* 61 677–685. 10.1016/j.neuroimage.2012.04.005 22521254

[B21] Disner S. G. Beevers C. G. Haigh E. A. P. Beck A. T. (2011). Neural mechanisms of the cognitive model of depression. *Nat. Rev. Neurosci.* 12 467–477. 10.1038/nrn3027 21731066

[B22] Eaton W. W. Badawi M. Melton B. (1995). Prodromes and precursors: epidemiologic data for primary prevention of disorders with slow onset. *Am. J. Psychiatry* 152 967–972. 10.1176/ajp.152.7.967 7793466

[B23] Eger E. Sterzer P. Russ M. O. Giraud A.-L. Kleinschmidt A. (2003). A supramodal number representation in human intraparietal cortex. *Neuron* 37 719–726. 10.1016/S0896-6273(03)00036-9 12597867

[B24] Elliott R. Rubinsztein J. S. Sahakian B. J. Dolan R. J. (2002). The neural basis of mood-congruent processing biases in depression. *Arch. Gen. Psychiatry* 59 597–604. 10.1001/archpsyc.59.7.597 12090812

[B25] Esslen M. Pascual-Marqui R. Hell D. Kochi K. Lehmann D. (2004). Brain areas and time course of emotional processing. *Neuroimage* 21 1189–1203. 10.1016/j.neuroimage.2003.10.001 15050547

[B26] Ferrari A. J. Charlson F. J. Norman R. E. Patten S. B. Freedman G. Murray C. J. (2013). Burden of depressive disorders by country, sex, age, and year: findings from the global burden of disease study 2010. *PLoS Med.* 10:e1001547 10.1371/journal.pmed.1001547 PMC3818162 24223526

[B27] First M. B. Spitzer R. L. Gibbon M. Williams J. B. (2001). *Structured Clinical Interview for DSM-IV-TR Axis I Disorders—Patient Edition (SCID-I/P. 2/2001 Revision).* New York, NY: New York State Psychiatric Institute.

[B28] Fitzgerald P. B. Laird A. R. Maller J. Daskalakis Z. J. (2008). A meta-analytic study of changes in brain activation in depression. *Hum. Brain Mapp.* 29 683–695. 10.1002/hbm.20426 17598168 PMC2873772

[B29] Foland-Ross L. Gotlib I. (2012). Cognitive and neural aspects of information processing in major depressive disorder: an integrative perspective. *Front. Psychol.* 3:489 10.3389/fpsyg.2012.00489 PMC3495336 23162521

[B30] Fu X. Taber-Thomas B. C. Pérez-Edgar K. (2017). Frontolimbic functioning during threat-related attention: relations to early behavioral inhibition and anxiety in children. *Biol. Psychol.* 122 98–109. 10.1016/j.biopsycho.2015.08.010 26325222 PMC4779741

[B31] Godlewska B. R. Browning M. Norbury R. Cowen P. J. Harmer C. J. (2016). Early changes in emotional processing as a marker of clinical response to SSRI treatment in depression. *Transl. Psychiatry* 6:e957 10.1038/tp.2016.130 PMC5314109 27874847

[B32] Gotlib I. H. Joormann J. Foland-Ross L. C. (2014). Understanding familial risk for depression a 25-year perspective. *Perspect. Psychol. Sci.* 9 94–108. 10.1177/1745691613513469 26173248 PMC11877285

[B33] Gotlib I. H. Krasnoperova E. Yue D. N. Joormann J. (2004). Attentional biases for negative interpersonal stimuli in clinical depression. *J. Abnorm. Psychol.* 113 121–135. 10.1037/0021-843X.113.1.121 14992665

[B34] Greening S. G. Osuch E. A. Williamson P. C. Mitchell D. G. (2014). The neural correlates of regulating positive and negative emotions in medication-free major depression. *Soc. Cogn. Affect. Neurosci.* 9 628–637. 10.1093/scan/nst027 23482626 PMC4014100

[B35] Greening S. G. Osuch E. A. Williamson P. C. Mitchell D. G. V. (2013). Emotion-related brain activity to conflicting socio-emotional cues in unmedicated depression. *J. Affect. Disord.* 150 1136–1141. 10.1016/j.jad.2013.05.053 23769293

[B36] Hamilton J. P. Etkin A. Furman D. J. Lemus M. G. Johnson R. F. Gotlib I. H. (2012). Functional neuroimaging of major depressive disorder: a meta-analysis and new integration of baseline activation and neural response data. *Am. J. Psychiatry* 169 693–703. 10.1176/appi.ajp.2012.11071105 22535198 PMC11889638

[B37] Hardee J. E. Benson B. E. Bar-Haim Y. Mogg K. Bradley B. P. Chen G. (2013). Patterns of neural connectivity during an attention bias task moderate associations between early childhood temperament and internalizing symptoms in young adulthood. *Biol. Psychiatry* 74 273–279. 10.1016/j.biopsych.2013.01.036 23489415 PMC3725217

[B38] Johnstone T. van Reekum C. M. Urry H. L. Kalin N. H. Davidson R. J. (2007). Failure to regulate: counterproductive recruitment of top-down prefrontal-subcortical circuitry in major depression. *J. Neurosci.* 27 8877–8884. 10.1523/JNEUROSCI.2063-07.2007 17699669 PMC6672169

[B39] Joormann J. Talbot L. Gotlib I. H. (2007). Biased processing of emotional information in girls at risk for depression. *J. Abnorm. Psychol.* 116 135–143. 10.1037/0021-843X.116.1.135 17324024

[B40] Kanske P. Heissler J. Schönfelder S. Wessa M. (2012). Neural correlates of emotion regulation deficits in remitted depression: the influence of regulation strategy, habitual regulation use, and emotional valence. *Neuroimage* 61 686–693. 10.1016/j.neuroimage.2012.03.089 22613776

[B41] Karsten J. Hartman C. A. Smit J. H. Zitman F. G. Beekman A. T. Cuijpers P. (2011). Psychiatric history and subthreshold symptoms as predictors of the occurrence of depressive or anxiety disorder within 2 years. *Br. J. Psychiatry* 198 206–212. 10.1192/bjp.bp.110.080572 21357879

[B42] Kellough J. L. Beevers C. G. Ellis A. J. Wells T. T. (2008). Time course of selective attention in clinically depressed young adults: an eye tracking study. *Behav. Res. Ther.* 46 1238–1243. 10.1016/j.brat.2008.07.004 18760771 PMC2584153

[B43] Kessler R. C. McGonagle K. A. Zhao S. Nelson C. B. Hughes M. Eshleman S. (1994). Lifetime and 12-month prevalence of DSM-III-R psychiatric disorders in the United States: results from the national comorbidity survey. *Arch. Gen. Psychiatry* 51 8–9. 10.1001/archpsyc.1994.03950010008002 8279933

[B44] Koster E. H. De Raedt R. Goeleven E. Franck E. Crombez G. (2005). Mood-congruent attentional bias in dysphoria: maintained attention to and impaired disengagement from negative information. *Emotion* 5 446–455. 10.1037/1528-3542.5.4.446 16366748

[B45] Lapate R. C. Rokers B. Tromp D. P. M. Orfali N. S. Oler J. A. Doran S. T. (2016). Awareness of emotional stimuli determines the behavioral consequences of amygdala activation and amygdala-prefrontal connectivity. *Sci. Rep.* 6:25826 10.1038/srep25826 PMC4867584 27181344

[B46] Lévesque J. Eugene F. Joanette Y. Paquette V. Mensour B. Beaudoin G. (2003). Neural circuitry underlying voluntary suppression of sadness. *Biol. Psychiatry* 53 502–510. 10.1016/S0006-3223(02)01817-6 12644355

[B47] Li H. Wei D. Browning M. Du X. Zhang Q. Qiu J. (2016). Attentional bias modification (ABM) training induces spontaneous brain activity changes in young women with subthreshold depression: a randomized controlled trial. *Psychol. Med.* 46 909–920. 10.1017/S003329171500238X 26554304

[B48] Ma Z. Li R. Yu J. He Y. Li J. (2013). Alterations in regional homogeneity of spontaneous brain activity in late-life subthreshold depression. *PLoS ONE* 8:e53148 10.1371/journal.pone.0053148 PMC3534624 23301035

[B49] MacLeod C. Rutherford E. Campbell L. Ebsworthy G. Holker L. (2002). Selective attention and emotional vulnerability: assessing the causal basis of their association through the experimental manipulation of attentional bias. *J. Abnorm. Psychol.* 111 107–123. 10.1037/0021-843X.111.1.107 11866165

[B50] Maldjian J. A. Laurienti P. J. Kraft R. A. Burdette J. H. (2003). An automated method for neuroanatomic and cytoarchitectonic atlas-based interrogation of fMRI data sets. *Neuroimage* 19 1233–1239. 10.1016/S1053-8119(03)00169-1 12880848

[B51] Miller C. H. Hamilton J. Sacchet M. D. Gotlib I. H. (2015). Meta-analysis of functional neuroimaging of major depressive disorder in youth. *JAMA Psychiatry* 72 1045–1053. 10.1001/jamapsychiatry.2015.1376 26332700 PMC11890701

[B52] Miller E. K. Cohen J. D. (2001). An integrative theory of prefrontal cortex function. *Annu. Rev. Neurosci.* 24 167–202. 10.1146/annurev.neuro.24.1.167 11283309

[B53] Mitterschiffthaler M. T. Williams S. C. R. Walsh N. D. Cleare A. J. Donaldson C. Scott J. (2008). Neural basis of the emotional Stroop interference effect in major depression. *Psychol. Med.* 38 247–256. 10.1017/S0033291707001523 17825123

[B54] Mogg K. Mathews A. May J. Grove M. Eysenck M. Weinman J. (1991). Assessment of cognitive bias in anxiety and depression using a colour perception task. *Cogn. Emot.* 5 221–238. 10.1080/02699939108411036

[B55] Monk C. Nelson E. McClure E. Mogg K. Bradley B. Leibenluft E. (2006). Ventrolateral prefrontal cortex activation and attentional bias in response to angry faces in adolescents with generalized anxiety disorder. *Am. J. Psychiatry* 163 1091–1097. 10.1176/ajp.2006.163.6.1091 16741211

[B56] Monk C. Telzer E. H. Mogg K. Bradley B. Mai X. Louro H. (2008). Amygdala and ventrolateral prefrontal cortex activation to masked angry faces in children and adolescents with generalized anxiety disorder. *Arch. Gen. Psychiatry* 65 568–576. 10.1001/archpsyc.65.5.568 18458208 PMC2443697

[B57] Naim R. Abend R. Wald I. Eldar S. Levi O. Fruchter E. (2015). Threat-related attention bias variability and posttraumatic stress. *Am. J. Psychiatry* 172 1242–1250. 10.1176/appi.ajp.2015.14121579 26206076 PMC6335584

[B58] Peckham A. D. McHugh R. K. Otto M. W. (2010). A meta-analysis of the magnitude of biased attention in depression. *Depress Anxiety* 27 1135–1142. 10.1002/da.20755 21049527

[B59] Pessoa L. McKenna M. Gutierrez E. Ungerleider L. (2002). Neural processing of emotional faces requires attention. *Proc. Natl. Acad. Sci. U.S.A.* 99 11458–11463. 10.1073/pnas.172403899 12177449 PMC123278

[B60] Roiser J. P. Elliott R. Sahakian B. J. (2011). Cognitive mechanisms of treatment in depression. *Neuropsychopharmacology* 37 117–136. 10.1038/npp.2011.183 21976044 PMC3238070

[B61] Shackman A. J. Salomons T. V. Slagter H. A. Fox A. S. Winter J. J. Davidson R. J. (2011). The integration of negative affect, pain and cognitive control in the cingulate cortex. *Nat. Rev. Neurosci.* 12 154–167. 10.1038/nrn2994 21331082 PMC3044650

[B62] Siegle G. J. Thompson W. Carter C. S. Steinhauer S. R. Thase M. E. (2007). Increased amygdala and decreased dorsolateral prefrontal BOLD responses in unipolar depression: related and independent features. *Biol. Psychiatry* 61 198–209. 10.1016/j.biopsych.2006.05.048 17027931

[B63] Smith K. (2014). Mental health: a world of depression. *Nature* 515:181 10.1038/515180a 25391942

[B64] Tamietto M. Cauda F. Celeghin A. Diano M. Costa T. Cossa F. M. (2015). Once you feel it, you see it: insula and sensory-motor contribution to visual awareness for fearful bodies in parietal neglect. *Cortex* 62 56–72. 10.1016/j.cortex.2014.10.009 25465122

[B65] Telzer E. H. Mogg K. Bradley B. P. Mai X. Ernst M. Pine D. S. (2008). Relationship between trait anxiety, prefrontal cortex, and attention bias to angry faces in children and adolescents. *Biol. Psychol.* 79 216–222. 10.1016/j.biopsycho.2008.05.004 18599179 PMC2574721

[B66] Tottenham N. Tanaka J. W. Leon A. C. McCarry T. Nurse M. Hare T. A. (2009). The NimStim set of facial expressions: judgments from untrained research participants. *Psychiatry Res.* 168 242–249. 10.1016/j.psychres.2008.05.006 19564050 PMC3474329

[B67] Townsend J. D. Bookheimer S. Y. Foland-Ross L. C. Moody T. D. Eisenberger N. I. Fischer J. S. (2012). Deficits in inferior frontal cortex activation in euthymic bipolar disorder patients during a response inhibition task. *Bipolar Disord.* 14 442–450. 10.1111/j.1399-5618.2012.01020.x 22631623 PMC4412746

[B68] Vuilleumier P. Armony J. L. Driver J. Dolan R. J. (2001). Effects of attention and emotion on face processing in the human brain: an event-related fMRI study. *Neuron* 30 829–841. 10.1016/S0896-6273(01)00328-2 11430815

[B69] Wager T. D. Davidson M. L. Hughes B. L. Lindquist M. A. Ochsner K. N. (2008). Neural mechanisms of emotion regulation: evidence for two independent prefrontal-subcortical pathways. *Neuron* 59 1037–1050. 10.1016/j.neuron.2008.09.006 18817740 PMC2742320

[B70] White L. K. Britton J. C. Sequeira S. Ronkin E. G. Chen G. Bar-Haim Y. (2016). Behavioral and neural stability of attention bias to threat in healthy adolescents. *Neuroimage* 136 84–93. 10.1016/j.neuroimage.2016.04.058 27129757 PMC5139370

[B71] Yoshimura S. Okamoto Y. Onoda K. Matsunaga M. Ueda K. Suzuki S.-I. (2010). Rostral anterior cingulate cortex activity mediates the relationship between the depressive symptoms and the medial prefrontal cortex activity. *J. Affect. Disord.* 122 76–85. 10.1016/j.jad.2009.06.017 19589603

[B72] Zhong M. Wang X. Xiao J. Yi J. Zhu X. Liao J. (2011a). Amygdala hyperactivation and prefrontal hypoactivation in subjects with cognitive vulnerability to depression. *Biol. Psychol.* 88 233–242. 10.1016/j.biopsycho.2011.08.007 21878364

[B73] Zhong M. Zhu X. Yi J. Yao S. Ruth Ann A. (2011b). Do the early attentional components of ERPs reflect attentional bias in depression? It depends on the stimulus presentation time. *Clin. Neurophysiol.* 122 1371–1381. 10.1016/j.clinph.2010.09.016 20961804

